# Impact of treatment adherence and inhalation technique on asthma outcomes of pediatric patients: a longitudinal study

**DOI:** 10.3389/fphar.2024.1340255

**Published:** 2024-03-13

**Authors:** Catalina Lizano-Barrantes, Olatz Garin, Karina Mayoral, Alexandra L. Dima, Angels Pont, María Araceli Caballero-Rabasco, Manuel Praena-Crespo, Laura Valdesoiro-Navarrete, María Teresa Guerra, Alberto Bercedo-Sanz, Montse Ferrer

**Affiliations:** ^1^ Health Services Research Group, Hospital del Mar Research Institute, Barcelona, Spain; ^2^ Department of Medicine and Life Sciences, Universitat Pompeu Fabra, Barcelona, Spain; ^3^ Department of Pharmaceutical Care and Clinical Pharmacy, Faculty of Pharmacy, Universidad de Costa Rica, San Jose, Costa Rica; ^4^ Centro de Investigación Biomédica en Red de Epidemiología y Salud Pública CIBERESP, Madrid, Spain; ^5^ National Heart and Lung Institute, Imperial College London, London, United Kingdom; ^6^ Health Technology Assessment in Primary Care and Mental Health (PRISMA), Institut de Recerca Sant Joan de Déu, Esplugues de Llobregat, Spain; ^7^ Pediatric Allergy and Pulmonology Unit, Pediatric Service, Hospital del Mar, Barcelona, Spain; ^8^ Centro de Salud La Candelaria, Servicio Andaluz de Salud, Seville, Spain; ^9^ Grupo de Vías Respiratorias de la Asociación Española de Pediatras de Atención Primaria (AEPAP), Madrid, Spain; ^10^ Pediatric Allergy and Pulmonology Unit, Pediatric Service, Hospital Universitari Parc Taulí, Sabadell, Spain; ^11^ Institut d’Investigació i Innovació Parc Taulí (I3PT-CERCA), Universitat Autònoma de Barcelona, Sabadell, Spain; ^12^ Centro de Salud Jerez Sur, Servicio Andaluz de Salud, Cadiz, Spain; ^13^ Centro de Salud Los Castros, Servicio Cántabro de Salud, Santander, Cantabria, Spain

**Keywords:** adherence, inhalation technique, pediatric asthma, health-related quality of life, asthma outcomes, asthma symptom control, asthma exacerbations

## Abstract

**Introduction:** We aimed to evaluate the longitudinal relationships, both at between- and within-person levels, that adherence to inhaled corticosteroid-based maintenance treatment and inhalation technique present with symptom control, exacerbations, and health-related quality of life (HRQoL) in children and adolescents with asthma.

**Methods:** Participants (6–14 years old) from the ARCA (Asthma Research in Children and Adolescents) cohort—a prospective, multicenter, observational study (NCT04480242)—were followed for a period from 6 months to 5 years via computer-assisted telephone interviews and a smartphone application. The Medication Intake Survey–Asthma (MIS-A) was administered to assess the implementation stage of adherence, and the Inhalation Technique Questionnaire (InTeQ) was used to assess the five key steps when using an inhaler. Symptom control was measured with the Asthma Control Questionnaire (ACQ), and HRQL was measured with the EQ-5D and the Patient-Reported Outcomes Measurement Information System–Pediatric Asthma Impact Scale (PROMIS-PAIS). Multilevel longitudinal mixed models were constructed separately with symptom control, exacerbation occurrence, EQ-5D, and PROMIS-PAIS as the dependent variables.

**Results:** Of the 360 participants enrolled, 303 (1,203 interviews) were included in the symptom control and exacerbation analyses, 265 (732) in the EQ-5D, and 215 (617) in the PROMIS-PAIS. Around 60% of participants were male subjects, and most of them underwent maintenance treatment with inhaled corticosteroids plus long-acting β-agonists in a fixed dose (73.3%). Within-person variability was 83.6% for asthma control, 98.6% for exacerbations, 36.4% for EQ-5D, and 49.1% for PROMIS-PAIS. At the within-person level, patients with higher adherence had better symptom control (*p* = 0.002) and HRQoL over time (*p* = 0.016). Patients with a better inhalation technique reported worse HRQoL simultaneously (*p* = 0.012), but they showed better HRQoL in future assessments (*p* = 0.012). The frequency of reliever use was associated with symptom control (*p* < 0.001), exacerbation occurrence (*p* < 0.001), and HRQoL (*p* = 0.042); and boys were more likely to present better symptom control and HRQoL than girls.

**Conclusion:** Our results confirm longitudinal associations at the within-person level of the two indicators of quality use of inhalers: for adherence to maintenance treatment with symptom control and HRQoL, and for the inhalation technique with HRQoL. Although treatment adherence was shown to be excellent, a third of the participants reported a suboptimal inhalation technique, highlighting the need for actions for improving asthma management of the pediatric population.

## 1 Introduction

Asthma is the most common non-communicable disease in school-aged children (Bercedo Sanz et al., 2022; The Global Asthma Report, 2022) and a major public health problem worldwide (Asher et al., 2021; Bercedo Sanz et al., 2022; Song et al., 2022). In 2019, an estimated 12,900 deaths occurred and 5.1 million disability-adjusted life years were lost due to childhood asthma (Zhang and Zheng, 2022). According to the latest global report, only 44.1% of children and 55.4% of adolescents with asthma achieved a well-controlled disease stage (García-Marcos et al., 2023).

Childhood asthma is a heterogeneous and fluctuating disease, with symptoms that vary in time and intensity (Global Initiative for Asthma; von Mutius and Smits, 2020). Therefore, management is mainly based on a continuous personalized cycle of assessment of asthma control (symptom control and risk factors for future exacerbations), any comorbidities that could contribute to symptom burden and poor health-related quality of life (HRQoL), and treatment (Global Initiative for Asthma, 2023). Intake of daily inhaled corticosteroids (ICS) is the currently recommended pharmacologic maintenance therapy in individuals of all ages (Montuschi and Barnes, 2011; National Heart Lung and Blood Institute (NHLBI), 2020; Global Initiative for Asthma, 2023). Research has shown that adherence to ICS and inhalation technique are dynamic and complex (Vrijens et al., 2016; Azzi et al., 2017; Almomani et al., 2021), with studies indicating generally low adherence to maintenance medication (20%–70%) (Herndon et al., 2012; Boutopoulou et al., 2018) and suboptimal inhalation technique (8%–22%) (Gillette et al., 2016) in children and/or adolescents.

Most evidence from systematic reviews suggests that whether it is children (Everhart and Fiese, 2009; Engelkes et al., 2015; Silva et al., 2015; Kocks et al., 2018; Usmani et al., 2018), adolescents (Engelkes et al., 2015; Silva et al., 2015; Kocks et al., 2018; Usmani et al., 2018; Vazquez-Ortiz et al., 2020; Roche et al., 2022), or adults (Bårnes and Ulrik, 2015; Engelkes et al., 2015; Kocks et al., 2018; Usmani et al., 2018; Vazquez-Ortiz et al., 2020; Roche et al., 2022), higher levels of adherence (Bårnes and Ulrik, 2015; Engelkes et al., 2015; Vazquez-Ortiz et al., 2020) and better inhalation technique (Kocks et al., 2018; Usmani et al., 2018; Roche et al., 2022), analyzed separately, are associated with better outcomes (symptom control, exacerbations, and/or HRQoL), although an inverse or null association has also been found (Bårnes and Ulrik, 2015; Engelkes et al., 2015; Kocks et al., 2018; Usmani et al., 2018; Vazquez-Ortiz et al., 2020; Roche et al., 2022). Furthermore, impaired HRQoL has also been linked with asthma-associated factors, such as severity (Everhart and Fiese, 2009), disease control, and exacerbations (Silva et al., 2015; Vazquez-Ortiz et al., 2020) in children, adolescents, and young adults.

These systematic reviews (Bårnes and Ulrik, 2015; Engelkes et al., 2015; Silva et al., 2015; Kocks et al., 2018; Usmani et al., 2018; Vazquez-Ortiz et al., 2020; Roche et al., 2022) show that more than 160 studies have been conducted involving patients with asthma that evaluated the relationships between adherence, inhalation technique, asthma control, asthma exacerbation, and/or HRQoL. However, only 22 of these studies included longitudinal analyses, with nine focusing exclusively on children and/or adolescents (Bukstein et al., 2007; Camargo et al., 2007; Delea et al., 2008; Hagmolen of ten Have et al., 2008; Lasmar et al., 2009; Elkout et al., 2012; Herndon et al., 2012; Krishnan et al., 2012; Tiggelman et al., 2015) and 13 encompassing adults as well (Osman et al., 1999; Balkrishnan and Christensen, 2000; McMahon et al., 2000; Stern et al., 2006; Santos et al., 2008; McNally et al., 2009; Smith et al., 2009; Mattke et al., 2010; Rohan et al., 2010; Sundell et al., 2011; Williams et al., 2011; Hyland et al., 2012; Yildiz et al., 2014). Notably, none of them considered the temporal stages of adherence (initiation, implementation, and discontinuation) described in 2012 (Vrijens et al., 2012). Although the systematic reviews were published between 2015 and 2022, none of the studies including longitudinal analyses, were conducted after 2013. Therefore, they probably do not reflect the Food and Drug Administration (FDA) requirement changes, contraindicating the use of long-acting beta-agonists (LABAs) without concurrent ICS (Chowdhury and Dal Pan, 2010).

The Global Asthma Initiative Guideline (GINA) (Global Initiative for Asthma, 2023) has continued to incorporate changes due to the collection of new evidence related to the efficacy and safety of ICS, LABA, and short-acting beta-agonists (SABAs). More recent longitudinal studies (Azzi et al., 2017; Johnson et al., 2017; Souverein et al., 2017; Papi et al., 2018; Dima et al., 2019; Vervloet et al., 2020; Vervloet et al., 2022; Hale et al., 2023; Sousa-Pinto et al., 2023) have presented further evidence of the long-term role of ICS adherence in asthma. However, none were conducted specifically on a pediatric population, only one included HRQoL (Hale et al., 2023), few specified the adherence stage considered (Souverein et al., 2017; Dima et al., 2019; Vervloet et al., 2020; Vervloet et al., 2022), and only one included medication adherence alongside the inhalation technique (Hale et al., 2023). These last two concepts are closely related, with the poor inhalation technique even being considered an unintentional form of adherence (van Boven et al., 2015), but they are usually identified as independent concepts (Monteiro et al., 2022). Two of the aforementioned systematic reviews (Engelkes et al., 2015; Kocks et al., 2018) have highlighted the scarcity of studies evaluating the impact of adherence and inhalation technique, assessed together, on asthma outcomes, despite the association that has been observed between them (Giraud et al., 2011; Maricoto et al., 2020).

A deeper insight into how adherence and inhaler technique evolve over time and affect the clinical outcomes and HRQoL in children could foster a ‘quality use of medications’ strategy (Braido et al., 2016), aligning with the current guidelines. Therefore, we aimed to evaluate the longitudinal relationships, both at between- and within-person levels, that adherence to ICS (alone or in combination with LABA) and inhalation technique present with symptom control, exacerbations, and HRQoL in children and adolescents with asthma.

## 2 Materials and methods

### 2.1 Study design and participants

Asthma Research in Children and Adolescents (ARCA) is a longitudinal, prospective, multicenter, observational study (NCT04480242) designed to provide evidence about the evolution of young patients with persistent asthma through regular follow-ups.

Patients were consecutively recruited from five outpatient pediatric pulmonology hospital units and nine primary care pediatric centers in Spain from January 2018 to March 2023 and were thus followed for a period from 6 months to 5 years. The inclusion criteria were as follows: aged 6–14, with a clinical diagnosis of asthma (history of characteristic symptoms and objective signs of variable airflow limitation) (Global Initiative for Asthma, 2017), undergoing treatment with ICS (alone or combined with LABA) for more than 6 months in the previous year, no concomitant respiratory diseases, and with access to a smartphone (their own or their parents’). Written informed consent was requested from the parents or legally authorized representatives of all participants, and additionally, oral consent was obtained from the children.

The participants were followed *via* the ARCA smartphone application (Mayoral et al., 2021) monthly and *via* computer-assisted telephone interviews (CATIs) performed by trained interviewers at enrollment, every 6 months (regular CATIs), and after each exacerbation (post-exacerbation CATIs). The ARCA application is available in three age versions: proxy response for children aged 6–7 years and self-response for participants aged 8–11 and ≥12 years. Through the application, participants reported any new exacerbations and completed the HRQoL instruments. Two versions of the CATIs were administered, one for parents or guardians of children under 8 years old (proxy response) and one for participants aged 8 and older (self-response). CATIs collected information on asthma symptom control, exacerbations, asthma treatments (maintenance and reliever), adherence to maintenance medication, inhalation technique, reliever use, and exacerbation occurrence for the period immediately before the interview. Demographic and clinical information was collected from medical records at enrollment.

For this analysis, we selected participants who had valid registries of at least two CATIs during a period with an ICS-based treatment prescribed for regular use (maintenance).

The ESPACOMP Medication Adherence Reporting Guideline (EMERGE) was followed (Adams N. P. et al., 2005).

### 2.2 Study variables

Medication information was collected at every CATI, including the active drug component, dose, type of inhaler device (pressurized metered-dose inhaler—pMDI and dry-powder inhaler—DPI) for the maintenance treatment, and the frequency of reliever medication use. Maintenance treatment was grouped into two categories: ICS in a fixed-dose combination with LABA (ICS plus LABA) and single ICS inhaler. Both categories were classified following the GINA preferred track steps, according to the ICS dose (low/medium/high) (Global Initiative for Asthma, 2023). The frequency of reliever medication use was measured with the following question: *How often have you usually taken your “reliever medication” (brand name) in the past 4 weeks: every day, almost every day, once or twice every week, or less than once a week?* This variable was grouped into the following: almost never (participants with no SABA prescribed and those reporting *used less than once a week*) and usually (participants reporting the first three response options).

Medication adherence was measured with the Medication Intake Survey–Asthma (MIS-A) (Dima et al., 2017), a validated instrument for telephone interviews, which assesses the implementation stage of adherence separately for each maintenance inhaler based on the self-reported prescription start date, daily dosage recommendations, and questions on maintenance use over increasing periods. Percentages of used *versus* prescribed medication are calculated first for each question and, subsequently, as composite scores. We used 1-month composite scores based on inhalations used the day before (Q1), days on which no inhalations were taken in the past 7 days (Q2), days on which all prescribed inhalations were used in the past 7 days (Q3), and days on which all prescribed inhalations were used in the past 28 days (Q4). MIS-A was administered at enrollment and at every 6 months in the regular CATIs and in the post-exacerbation CATIs. When patients used more than one inhaler containing ICS, we computed scores for each inhaler and averaged across them. MIS-A has been validated (Dima et al., 2017) using self-response in adult patients and teenagers and a proxy version for the caregivers of children in English and French. The MIS-A was linguistically adapted into Spanish for the pediatric population within the ARCA study, according to the recommended methodology (double direct translation, translation synthesis, back-translation, and cognitive debriefing) (Wild et al., 2005).

The inhalation technique was measured with the Inhaler Technique Questionnaire (InTeQ) (Lizano-Barrantes et al., 2022; Lizano-Barrantes et al., 2023a), an instrument that assesses the frequency of performing five key steps when using the inhaler in the previous 6 months with a five-level Likert scale (from “always” to “never”). The InTeQ was administered in the CATIs at enrollment and yearly. A global score was calculated as a sum of the InTeQ items answered “always,” among the four, which demonstrated unidimensionality in children and adolescents (Lizano-Barrantes et al., 2023a), and was categorized into the following: 4–3 (good inhaler technique), 2 (fair), and 1–0 (poor). The InTeQ has been validated for telephone interviews (Lizano-Barrantes et al., 2023a) using self-response in children aged 8 and older and proxy response for parents or guardians of children under 8 years old. As the InTeQ was only administered yearly, the missing values were replaced by data from the previous interview.

Symptom control was measured with the asthma control questionnaire (ACQ)– symptoms only (Juniper et al., 2005a), which was administered in the regular and post-exacerbation CATIs. It assesses the presence and intensity of night-time waking, symptoms on waking, activity limitation, shortness of breath, and wheezing during the previous week on a 7-level Likert scale from 0 (no impairment) to 6 (maximum impairment). The overall score, calculated as the mean item responses, ranges from 0 to 6. Cut-off points of 1.5 and 0.75 were established to define not well- and well-controlled asthma, respectively (Juniper et al., 2006). The ACQ has been validated (Juniper et al., 2005b) using self-administration in adolescents and interviewer administration in children.

Asthma exacerbations were identified in the regular CATIs administered every 6 months or by reporting them through the application, which prompted an alert to the research team that was followed by a post-exacerbation CATI to confirm its occurrence. In both cases, exacerbations were defined through three questions that were constructed applying the definitions by the American Thoracic Society and the European Respiratory Society (Reddel et al., 2009): *Did you visit or phone your family doctor or outpatient emergency department because your asthma got worse? Did you call an ambulance or go to the hospital because of your asthma? Did you take steroid tablets or syrup (such as prednisolone or Deltacortril) for at least 2 days because of your asthma?* If the participant answers “yes” to at least one of the three questions, an asthma exacerbation is confirmed.

Health-related quality of life (HRQoL) was measured using two complementary instruments, the EuroQol generic questionnaire (EQ-5D) (Ravens-Sieberer et al., 2010; Wille et al., 2010; Gusi et al., 2014) and the disease-specific questionnaire Patient-Reported Outcomes Measurement Information System–Pediatric Asthma Impact Scale (PROMIS-PAIS) (Yeatts et al., 2010), which were administered through the ARCA application. The EQ-5D was administered at enrollment and every 6 months. It consists of five dimensions, namely, “mobility,” “looking after myself,” “doing usual activities,” “having pain/discomfort,” and “feeling worried/sad/unhappy, with a time frame of “today.” According to the age, we used the EQ-5D-Y-3L proxy-version (6–7 years), the self-administered EQ-5D-Y-3L (8–11 years), and the self-administered EQ-5D-5L (≥12 years). A single preference-based utility index was calculated ranging from 1 (the best health state) to negative values (health states valued by society as worse than death), where 0 is equal to death. Preference value sets applied to generate this utility index were those obtained from Spanish adults for the EQ-5D-5L (Ramos-Goñi et al., 2018) and those obtained from Spanish adults thinking as a hypothetical 10-year-old child for the EQ-5D-Y (Ramos-Goñi et al., 2020; Ramos-Goñi et al., 2024). The short form 8a version of the PROMIS-PAIS (v2.0) was administered at 4 months from enrollment and at every 6 months thereafter. Its items ask about the past 7 days in a 5-level Likert response scale (1–5) with the following options: never, almost never, sometimes, often, and almost always. It is available for self-response for ages 8–17 and for proxy response for children starting at age 5. The total raw score is calculated by adding the values of the response to each question, ranging from 8 to 40 (a lower score indicates better HRQoL) (A brief guide to the PROMIS, 2023).

### 2.3 Analytical strategy

To specifically examine the impact of the implementation stage of adherence to an ICS-based maintenance treatment (i.e., the degree to which patients follow their prescribed doses during treatment), we censored from the dataset reports under certain conditions: no prescribed daily ICS at all, ICS prescribed on an as-needed basis, or prescribed other asthma maintenance treatment (such as tiotropium). Descriptive analyses were performed of patients’ follow-up, reports, patient characteristics, treatment, and outcomes by calculating the percentages or means and standard deviations. Differences between the patients included and not included in the analysis of each outcome (asthma symptom control, exacerbation, EQ-5D, and PROMIS-PAIS) were assessed with a chi-squared or *t*-test, according to the type of variable.

Continuous time-varying predictors (adherence and the inhalation technique) were decomposed into three variables to distinguish the between-person effects and the simultaneous and sequential within-person effects. Average adherence was calculated as the mean score for each patient across all reports (one score per patient) and used for examining whether differences in adherence between patients predict the outcomes. Current fluctuation was computed as the difference between a patient’s average adherence and their score in a given report (multiple scores per patient) to examine whether changes in adherence within patients are associated with concomitant changes in the outcome (i.e., measured in the same report). Prior fluctuation was computed as a lagged variable, i.e., the difference between a patient’s average and the score in their previous report, usually 6 months earlier (multiple scores per patient), to examine whether changes in adherence predict the outcomes measured in the subsequent report.

To assess longitudinal relationships of adherence to ICS-based maintenance treatment and the inhalation technique with outcomes, we followed established procedures for hierarchical longitudinal modeling (Singer and Willett, 2003). Four multilevel longitudinal mixed models were constructed separately for asthma symptom control, exacerbation occurrence, EQ-5D, and PROMIS-PAIS (as dependent variables). In all cases, models were constructed to assess the role of the two time-varying variables, adherence and inhalation technique (which are the main explanatory variables), including them together with the type of ICS-based maintenance treatment and sociodemographic variables that can be potential confounders (model A); then, other factors that are part of the implicit standard for asthma management were added (Dima et al., 2016; Global Initiative for Asthma, 2023), namely, the use of a reliever, asthma symptom control, and the occurrence of exacerbations, except in models where they were the dependent variables (model B). Time was modeled as years since the first interview per patient (random and fixed), and interactions between the independent variables and time were tested. In addition to the *p*-values of each coefficient or odds ratio (OR) provided by the models, ANOVA was applied to test the significance corresponding to each independent variable. Sensitivity analyses were performed with 1-week adherence scores.

R (version 4.2.2) and RStudio (2022.07.2 Build 576) were used to construct all the models, except for the exacerbation occurrence, which was constructed with SAS 9.4.

## 3 Results

**FIGURE 1:**
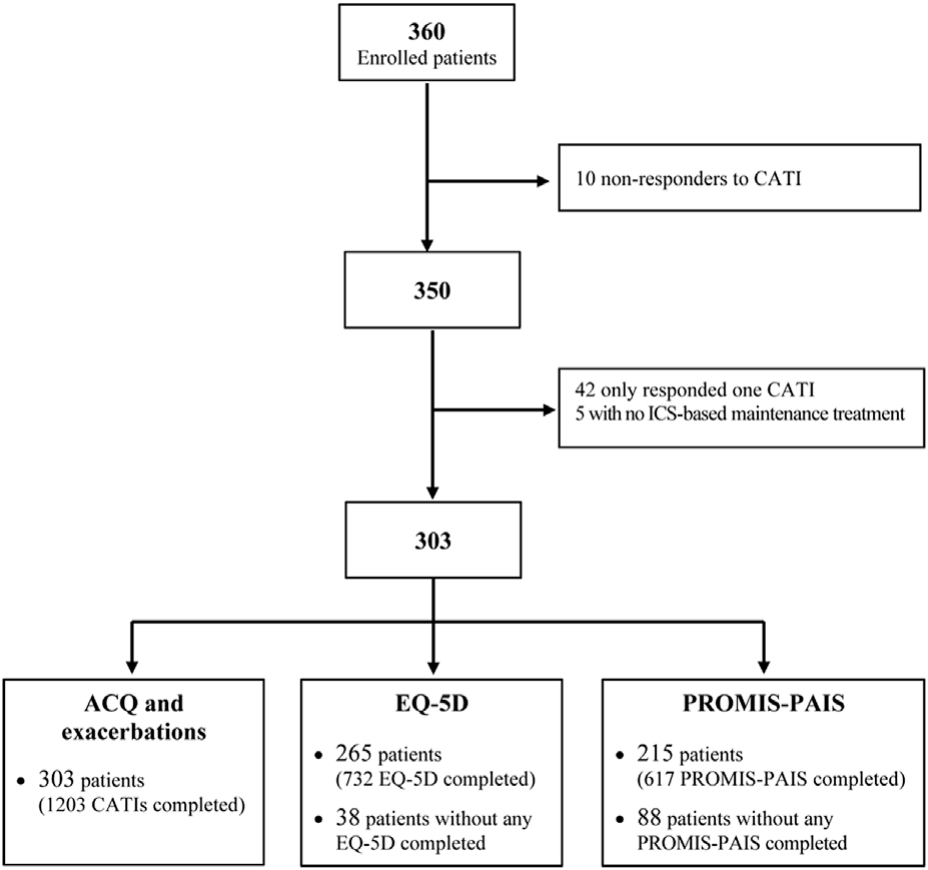
Flowchart of the selection of patients meeting the analysis criteria. CATI: computer-assisted telephone interview; ICS: inhaled corticosteroids; ACQ: Asthma Control Questionnaire; PROMIS-PAIS: Patient-Reported Outcomes Measurement Information System–Pediatric Asthma Impact Scale.

Out of the 360 participants enrolled from January 2018 to March 2023 (Figure 1), we excluded the following from the analysis: 10 who did not respond to any CATI, 42 with only one valid CATI, and 5 without ICS-based maintenance treatment. Then, 303 valid participants (who responded to a total of 1,203 CATIs) were included in the analysis of asthma symptom control and exacerbation, 265 participants (with 732 questionnaires completed) in the EQ-5D analysis, and 215 (with 617 questionnaires completed) in the analysis of PROMIS-PAIS. Globally, patients provided 2–9 reports (Q2 (median) = 4, Q1-Q3 = 2–5), with a mean (SD) follow-up of 692 (419) days (range 116–1,759 days) in the analyses of symptom control and exacerbation occurrence. For the EQ-5D and PROMIS-PAIS analyses, reports per patient ranged from 1 to 8, with medians of 2 (Q1–Q3 = 2–4) and 3 (Q1–Q3 = 2–4), respectively.

**FIGURE 2:**
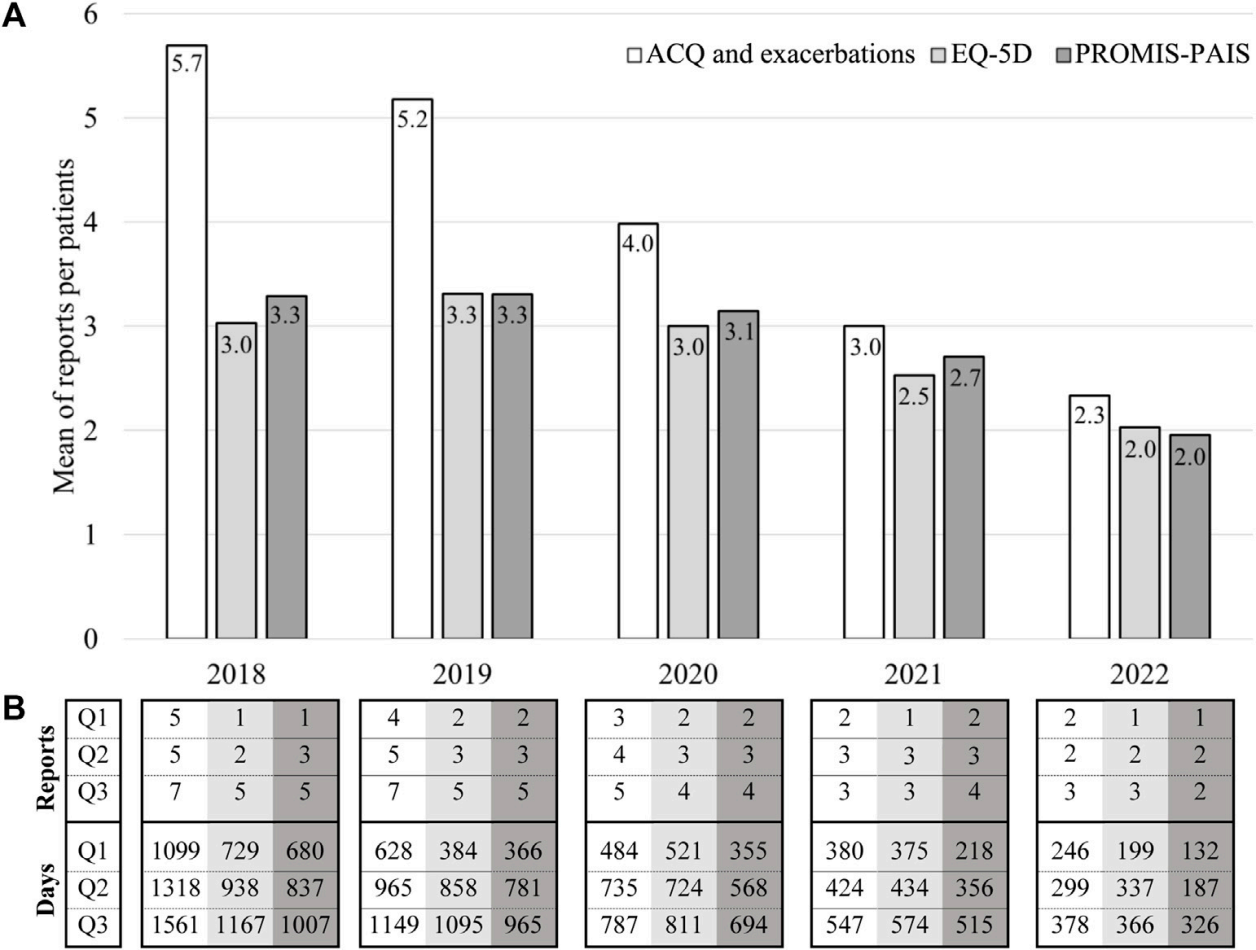
Description of patients, reports, and days analyzed by the year of follow-up initiation. (**(A)**-bar chart) Number of patients by the year of follow-up initiation. (**(B)**-table) Number of reports and days analyzed per patient by the year of follow-up initiation. n: number of patients; %: percentage of patients included in the EQ-5D and PROMIS-PAIS analyses relative to those included in the ACQ and exacerbation analyses; Q1: percentile 25; Q2: percentile 50 or median; Q3: percentile 75.

Figure 2A shows the number of patients who started follow-up in each year and were valid for analysis. For example, of the patients followed since 2018 in the ARCA cohort, 43 were included in the analysis of symptom control and exacerbation occurrence (white bar), 34 in the EQ-5D one (light gray bar), and 28 in the PROMIS-PAIS (dark gray bar) analysis. Enrollment for the study peaked in 2019 and then faced challenges in 2020 due to the SARS-CoV-2 pandemic, but it was sustained through effective mitigation efforts. The mean number of reports completed per patient is shown in Figure 2B. For instance, the 43 patients who started follow-up in 2018 provided a mean of 5.7 reports, while the 66 patients who started follow-up in 2022 provided 2.3 reports on average. These differences in the number of valid reports per participant are due to the duration of the follow-up, according to the year of enrollment, which was 1,318 vs. 299 days of median (Q2) for patients followed since 2018 and 2022, respectively, in the analyses of symptom control and exacerbation occurrence.

The characteristics of the participants are presented in Table 1. The majority were male subjects (60.7%), reported using relievers less than once a week (55.4%), undergoing maintenance treatment with ICS combined with LABA in a fixed dose (73.3%), administered by pMDI (74.6%), and similarly distributed among steps 2–3 (33.0%), step 4 (37.4%), and step 5 (29.5%) of the GINA preferred track, according to the dose of ICS. The mean 1-month adherence score was 87.8%; 45.2% of participants reported a good inhalation technique, and 64.2% had well-controlled symptoms. Experiencing exacerbations were reported by 37.8% of the participants. The HRQoL score measured with the EQ-5D was 0.93 (1, best health state to negative values, worse than death), and when measured with the PROMIS-PAIS, it was 13.0 (8, best health state to 40, worst).

**TABLE 1:** Demographic and clinical characteristics of the participants.

		All (n = 350)	ACQ and exacerbations (n = 303)	EQ-5D (n = 265)	PROMIS-PAIS (n = 215)
Sex, n (%)	Male	216 (61.7%)	184 (60.7%)	160 (60.4%)	130 (60.5%)
	Female	134 (38.3%)	119 (39.3%)	105 (39.6%)	85 (39.5%)
	*p*-value		0.334	0.364	0.544
Age, n (%)	6–7 years	86 (24.6%)	73 (24.1%)	65 (24.5%)	52 (24.2%)
	8–11 years	166 (47.4%)	147 (48.5%)	131 (49.4%)	110 (51.2%)
	≥12 years	98 (28.0%)	83 (27.4%)	69 (26.0%)	53 (24.7%)
	*p*-value		0.586	0.297	0.142
Maintenance treatment, n (%)	ICS	101 (28.9%)	81 (26.7%)	72 (27.2%)	56 (26.0%)
	ICS plus LABA	241 (68.9%)	222 (73.3%)	193 (72.8%)	159 (74.0%)
	Other treatment	7 (2.0%)	0 (0.0%)	0 (0.0%)	0 (0.0%)
	No treatment	1 (0.3%)	0 (0.0%)	0 (0.0%)	0 (0.0%)
	*p*-value		<0.001	<0.001	0.001
Type of inhaler device, n (%)	pMDI	253 (74.0%)	226 (74.6%)	207 (78.1%)	170 (79.1%)
	DPI	67 (19.6%)	59 (19.5%)	53 (20.0%)	40 (18.6%)
	Unknown	22 (6.4%)	18 (5.9%)	5 (1.9%)	5 (2.3%)
	*p*-value		0.562	<0.001	<0.001
GINA step, n (%)	Step 2–3 (low-dose ICS)	93 (33.0%)	82 (32.7%)	43 (32.2%)	64 (33.7%)
	Step 4 (medium-dose ICS)	105 (37.4%)	93 (37.1%)	86 (37.9%)	68 (35.8%)
	Step 5 (high-dose ICS)	83 (29.5%)	76 (30.3%)	68 (30.0%)	58 (30.5%)
	*p-value*		0.730	0.791	0.724
Reliever use, n (%)	Not prescribed	22 (6.3%)	19 (6.3%)	18 (6.8%)	14 (6.5%)
	Less than once a week	194 (55.6%)	168 (55.6%)	147 (55.7%)	114 (53.3%)
	Once or twice a week	90 (25.8%)	80 (26.5%)	70 (26.5%)	60 (28.0%)
	Almost every day	29 (8.3%)	25 (8.3%)	23 (8.7%)	21 (9.8%)
	Every day	14 (4.0%)	10 (3.3%)	6 (2.3%)	5 (2.3%)
	*p-value*		0.648	0.094	0.175
% Last month adherence, mean (SD)		86.7 (23.3)	87.8 (21.3)	88.0 (21.0)	88.4 (20.8)
	*p*-value		0.029	0.056	0.084
% Last week adherence, mean (SD)		85.0 (27.2)	85.8 (25.9)	86.7 (25.1)	86.4 (25.5)
	*p*-value		0.125	0.032	0.198
Inhalation technique, n (%)	Poor (0–1 always)	106 (31.7%)	95 (32.5%)	83 (32.0%)	65 (31.0%)
	Fair (2 always)	81 (24.3%)	65 (22.3%)	56 (21.6%)	48 (22.9%)
	Good (3–4 always)	147 (44.0%)	132 (45.2%)	120 (46.3%)	97 (46.2%)
	*p*-value		0.082	0.094	0.556
ACQ	Not well-controlled (>1.5)	71 (20.9%)	63 (21.3%)	51 (19.5%)	40 (18.9%)
	Intermediate (0.75–1.5)	48 (14.2%)	43 (14.5%)	40 (15.3%)	35 (16.5%)
	Well-controlled (<0.75)	220 (64.9%)	190 (64.2%)	171 (65.3%)	137 (64.6%)
	*p*-value		0.767	0.328	0.185
Exacerbation, n (%)	No	210 (61.9%)	184 (62.2%)	161 (61.5%)	134 (63.2%)
	Yes	129 (38.1%)	112 (37.8%)	101 (38.5%)	78 (36.8%)
	*p*-value		0.830	0.728	0.537
EQ-5D, mean (SD)				0.93 (0.11)	
PROMIS, mean (SD)					13.0 (5.7)

*p*-values assessing differences between the patients included and those not included in each subsample corresponding to the chi-squared test or *t*-test, according to the type of variable.

Table 2 shows the results for the longitudinal associations that maintenance treatment adherence and the inhalation technique present with asthma symptom control (left column) and exacerbations (right column). The proportion of between-person variation was 16.4% for asthma control and 1.4% for exacerbations. Model A with asthma symptom control shows that, at the within-person level, patients reporting higher adherence to maintenance medication also reported better control in the next interview (prior fluctuation; *p* = 0.002). On the contrary, both models A and B show that girls (*p* = 0.006 and *p* = 0.012) were more likely to report worse control of asthma symptoms. Furthermore, model B shows that patients who reported using reliever medication ≥1–2 times per week (*p* < 0.001) and having an exacerbation (*p* < 0.001) were also more likely to present uncontrolled asthma symptoms. Age, the inhalation technique, and the type of maintenance treatment (ICS alone or in combination with LABA) did not present any statistically significant association with asthma symptom control.

**TABLE 2:** Multilevel models of asthma symptom control (linear) and exacerbation occurrence (logistic).

	Asthma symptom control		Exacerbation occurrence	
	Model A	Model B	Model A	Model B
	b (SE)		OR (SE)	
Intercept	0.281 (0.253)^§^	0.069 (0.203)^§^	0.45 (0.60)	0.81 (0.69)
Time (years)	0.000 (0.003)	0.000 (0.002)	0.97 (0.01) ***^§^	0.97 (0.01) ***^§^
ADHERENCE				
Average adherence	0.001 (0.003)	0.001 (0.002)	1.00 (0.01)	0.99 (0.01)
Current fluctuation of adherence	−0.002 (0.002)	−0.002 (0.001)	0.99 (0.00)	0.99 (0.00)
Prior fluctuation of adherence	−0.005 (0.002) **^§^	−0.002 (0.001)^§^	0.99 (0.00)	0.99 (0.01)
INHALATION TECHNIQUE				
Average IT	0.044 (0.040)	0.016 (0.032)	1.21 (0.10)	1.16 (0.10)
Current fluctuation of IT	0.002 (0.036)	−0.009 (0.033)	1.10 (0.12)	1.06 (0.12)
Prior fluctuation of IT	−0.032 (0.040)	−0.046 (0.036)	1.14 (0.13)	1.11 (0.14)
Treatment				
ICS plus LABA	Ref.	Ref.	Ref.	Ref.
ICS	0.120 (0.091)	0.073 (0.074)	1.06 (0.23)	0.97 (0.24)
Sex				
Male	Ref.^§^	Ref.^§^	Ref.	Ref.
Female	0.233 (0.085) **	0.168 (0.067) *	1.06 (0.20)	0.89 (0.21)
Age				
<8 years	Ref.	Ref.	Ref.^§^	Ref.^§^
8–11	−0.097 (0.104)	−0.055 (0.083)	0.44 (0.24) ***	0.42 (0.25) ***
≥12	−0.053 (0.119)	−0.009 (0.094)	0.38 (0.28) ***	0.34 (0.30) ***
Reliever use				
Almost never		Ref.		Ref.^§^
Usually		0.840 (0.063) ***^§^		3.27 (0.23) ***
Exacerbation				
No		Ref.		
Yes		0.354 (0.067) ***^§^		
Asthma symptom control				
Not well-controlled				Ref.^§^
Intermediate				0.42 (0.38) *
Well-controlled				0.47 (0.29) **
ICC (linear); VPC (logistic)	0.2568	0.1641	0.0129	0.0142
Log-likelihood	−1,085.3	−978.8		
AIC	2,200.6	1,991.5		
BIC	2,271.0	2,071.4		
−2 Res Log Pseudo-Likelihood			3,726.66	3,838.27
Generalized chi-square			678.05	665.65
Generalized chi-square/DF			0.87	0.86

The *p*-values corresponding to each coefficient or OR, provided by the models were marked with asterisks: *(*p* < 0.05), **(*p* < 0.01), and ***(*p* < 0.001).

ANOVA *p*-values for each independent variable were marked with § (*p* < 0.05).

Exacerbations models A and B show less risk of occurrence in children aged 8 years or older (*p* ≤ 0.001 in both models) and participants reporting better asthma symptom control (*p* = 0.023 and *p* = 0.008). Conversely, the risk of exacerbation occurrence is higher in participants reporting using reliever medication ≥1–2 times per week (*p* < 0.001). Neither average adherence and the inhalation technique nor their prior or simultaneous fluctuations were associated with exacerbation occurrence.

The proportion of between-person variation was 63.6% and 50.9% for HRQoL (Table 3), EQ-5D, and PROMIS-PAIS, respectively. The EQ-5D models reveal that when participants reported a better inhalation technique, they reported worse HRQoL simultaneously (current fluctuation; *p* = 0.012 and *p* = 0.012), but they also reported better HRQoL in the next interview (prior fluctuation; *p* = 0.005 and *p* = 0.012). Furthermore, worse HRQoL was more likely in girls (*p* = 0.037 and *p* = 0.036). Age, adherence, type of treatment, the use of reliever medication, and the occurrence of exacerbations were not statistically significantly associated with EQ-5D.

**TABLE 3:** Multilevel models of health-related quality of life measured with the EQ-5D and PROMIS-PAIS (linear).

	EQ-5D		PROMIS-PAIS	
	Model A	Model B	Model A	Model B
	b (SE)		b (SE)	
Intercept	0.986 (0.044) ***^§^	0.988 (0.046) ***^§^	11.264 (2.384) ***^§^	11.988 (2.397) ***^§^
Time (years)	0.000 (0.001)	0.000 (0.001)	−0.034 (0.028)	0.038 (0.140)
ADHERENCE				
Average adherence	0.0004 (0.000)	0.0004 (0.000)	0.027 (0.024)	0.031 (0.023)
Current fluctuation of adherence	0.0002 (0.000)	0.0002 (0.000)	0.007 (0.012)	0.010 (0.012)
Prior fluctuation of adherence	0.0004 (0.000)	0.0002 (0.000)	−0.023 (0.012)	−0.009 (0.013)
INHALATION TECHNIQUE				
Average IT	0.002 (0.007)	0.003 (0.006)	−0.624 (0.379)	−0.658 (0.352)
Current fluctuation of IT	−0.015 (0.006) *^§^	−0.015 (0.006) *^§^	−0.316 (0.328)	−0.244 (0.324)
Prior fluctuation of IT	0.021 (0.007) **^§^	0.019 (0.007) *^§^	−0.231 (0.364)	−0.094 (0.359)
Treatment				
ICS plus LABA	Ref.	Ref.	Ref.	Ref.
ICS	−0.012 (0.016)	−0.011 (0.016)	−0.074 (0.902)	−0.041 (0.847)
Sex				
Male	Ref.^§^	Ref.^§^	Ref.^§^	Ref.^§^
Female	−0.029 (0.014) *	−0.029 (0.014) *	2.538 (0.824) **	2.457 (0.764) **
Age				
<8 years	Ref.	Ref.	Ref.^§^	Ref.^§^
8–11	0.010 (0.017)	0.006 (0.017)	−0.395 (0.979)	0.073 (0.918)
≥12	0.005 (0.020)	0.001 (0.020)	2.383 (1.169) *	2.709 (1.093) *
Asthma symptom control				
Not well-controlled		Ref.^§^		Ref.^§^
Intermediate		−0.018 (0.019)		0.305 (1.053)
Well-controlled		0.016 (0.014)		−2.403 (0.825) **
Reliever use				
Almost never		Ref.		Ref.
Usually		−0.012 (0.012)		1.319 (0.646) *
Exacerbation				
No		Ref.		Ref.
Yes		−0.014 (0.011)		0.082 (0.616)
Interaction time * Adherence last month				
Average adherence				−0.001 (0.002)
Current fluctuation of adherence				0.001 (0.001)
Prior fluctuation of adherences				−0.003 (0.001) *^§^
ICC	0.6161	0.6362	0.5716	0.5090
Log-likelihood	312.7	304.6	−1,386.4	−1,400.3
AIC	−591.4	−567.3	2838.2	2848.6
BIC	−522.5	−482.3	2907.7	2946.3

The *p*-values corresponding to each coefficient provided by the models were marked with asterisks: *(*p* < 0.05), **(*p* < 0.01), and ***(*p* < 0.001).

ANOVA *p*-values for each independent variable were marked with § (*p* < 0.05).

In PROMIS-PAIS models, the interaction between time and adherence reveals an increase in HRQoL over time, correlating with higher levels of patient-reported adherence in subsequent interviews (prior fluctuation; *p* = 0.016). Furthermore, better asthma symptom control was also associated with better HRQoL (*p* = 0.004). Conversely, worse HRQoL was more likely for adolescents compared to children under 12 years of age (*p* = 0.043 and *p* = 0.014), girls (*p* = 0.002 and *p* = 0.002), and the use of reliever medication ≥1–2 times a week (*p* = 0.042). The type of maintenance treatment regimen, the inhalation technique, and exacerbation did not present a statistically significant association with PROMIS-PAIS.

Sensitivity analysis with 1-week adherence scores showed similar results (Supplementary Material).

## 4 Discussion

This study provides evidence regarding the longitudinal relationships that maintenance treatment adherence and the inhaler technique present with asthma symptom control, exacerbations occurrence, and HRQoL in pediatric asthma patients. We gathered comprehensive patient-reported data using a combination of the ARCA application and CATIs. We found that better asthma symptom control over time (future assessments) was more likely in patients with higher adherence to treatment, while boys and those participants who reported almost never using reliever medication or no exacerbations generally had better symptom control. In the same direction, a lower risk of exacerbations was found in older children, those reporting well-controlled symptoms, and in those who almost never used reliever medication. Better HRQoL over time was observed in patients who reported better adherence and inhalation technique. Additionally, boys and participants with better symptom control generally had better HRQoL.

### 4.1 Adherence to ICS-based maintenance treatment

The level of adherence to maintenance treatment in ARCA participants is high on average; they reported having administered 88% of the prescribed dose during the previous month, which is above the range of 20%–70% identified by a systematic review (Herndon et al., 2012; Boutopoulou et al., 2018) in children and/or adolescents.

Consistent with our hypothesis, we found that higher adherence was associated with better asthma symptom control in future assessments, despite the inconsistent results reported both by systematic reviews (Gillette et al., 2016; Vazquez-Ortiz et al., 2020), which mainly included cross-sectional studies, and by more recent longitudinal studies (Dima et al., 2019; Vervloet et al., 2020; Vervloet et al., 2022; Sousa-Pinto et al., 2023). Consistently with our finding, a longitudinal study in French and English adults and children with asthma (Dima et al., 2019) showed that patients maintaining high ICS adherence over time have better asthma control. In the same line, a study of the large Nivel Primary Care Database in the Netherlands shows an association between poor ICS adherence and uncontrolled asthma (Papi et al., 2018). Conversely, a United Kingdom study (Vervloet et al., 2020) using the Optimum Patient Care Research Database (OPCRD) found that patients might adjust their ICS based on the current needs without this necessarily impacting later in hospitalizations, emergency visits, outpatient visits, or the need for oral corticosteroids or antibiotics. Additionally, a longitudinal study in patients from 27 countries with ICS plus LABA maintenance treatment pointed out that most patients only use medication when they are not well (Sousa-Pinto et al., 2023). Overall, these findings lead us to incorporate nuances into our hypothesis: the association between adherence and asthma control might be driven by an increased adherence as a reactive response to uncontrolled symptoms, which could eventually lead to increased symptom control over time.

The association found between increased treatment adherence and increased HRQoL over time is also consistent with our hypotheses as it could reflect an individual’s overall investment in maintaining their health and well-being through effective asthma management practices. This association was particularly identified with the asthma-specific questionnaire PROMIS-PAIS, likely due to its focused content, which is potentially more responsive to asthma symptoms (Wiebe et al., 2003). Although the specific association of adherence with HRQoL has been less frequently examined, our results are consistent with findings of a systematic review in adolescents (Usmani et al., 2018) and a multicenter, observational, prospective study in Greek adults with variable asthma severity (Exarchos et al., 2022). Additionally, a longitudinal study in Dutch adolescents (Tiggelman et al., 2015) indicated that higher HRQoL at baseline predicted increased medication adherence at follow-up, although good medication adherence did not predict an increase in HRQoL over time. These results line up with our enhanced hypothesis, distinguishing patients with regular adherence who actively integrate treatment into their daily routines, recognizing its importance, from those with “reactive adherence” who strictly follow treatments only when they feel that their asthma is out of control.

Although there is a substantial body of evidence from RCTs (Adams N. et al., 2005; Adams N. P. et al., 2005; Pauwels et al., 2003; O’byrne et al., 2001) showing that ICS-based maintenance treatment reduces exacerbation risks, there is less consistency in its association with adherence to this type of treatment. Our findings indicate a lack of association between adherence and exacerbation occurrence, which were consistent with observations from the abovementioned longitudinal studies in France, the United Kingdom, and the Netherlands (Dima et al., 2019; Vervloet et al., 2020; Vervloet et al., 2022), and a meta-analysis centered on the effect of interventions to improve adherence to ICS-based maintenance treatments, indicating that they may not always correlate with enhanced clinical outcomes (Normansell et al., 2017). Nevertheless, they contrast with a meta-analysis showing the association between treatment adherence and severe asthma exacerbations (Chongmelaxme et al., 2020). On one hand, it is important to highlight that response bias cannot be discarded in our study since interviews were performed immediately after experiencing an exacerbation, which could have made patients feel accountable, i.e., the patient’s behavior may be influenced by the expectation of social interactions with healthcare providers (Oussedik et al., 2017). On the other hand, taking into account that almost 70% of the participants in our study received a medium or high ICS dose, some of them may be candidates for a step-up in treatment, as suggested by a United Kingdom large cohort of adult patients in GINA step 3 or 4 of asthma management (Papi et al., 2018).

### 4.2 Inhalation technique

In our study, 32% of participants reported poor inhalation technique, which is above the proportion of the suboptimal inhalation technique reported by the studies of children and/or adolescents with asthma (8%–22%) identified in a systematic review (Gillette et al., 2016).

Given the recognized importance of both inhalation technique and adherence in impacting actual drug exposure (Global Initiative for Asthma, 2023; Ramos-Goñi et al., 2024), we hypothesized finding a similar association when both factors were analyzed together. Our results focusing on within-person fluctuations of the inhalation technique revealed that when participants temporarily improved their technique, their HRQoL decreased during that same period, but it improved afterward. This is also consistent with our hypothesis distinguishing between regular and reactive behaviors, suggesting similar patterns for inhalation technique and adherence, where a proactive approach to asthma management, even if initially challenging, ultimately contributes to enhanced HRQoL. These fluctuations are likely due to factors changing within patients with asthma over time rather than stable differences between patients, as highlighted in the longitudinal study involving French and English adults and children (Dima et al., 2019).

Three systematic reviews (Kocks et al., 2018; Usmani et al., 2018; Roche et al., 2022) supported that better inhalation technique, analyzed without considering adherence, are consistently associated with exacerbations (Usmani et al., 2018; Roche et al., 2022) and HRQoL (Kocks et al., 2018; Usmani et al., 2018; Roche et al., 2022), but there are less consistent results with asthma symptoms control (Kocks et al., 2018; Usmani et al., 2018; Roche et al., 2022). However, evidence on the relationship between inhalation technique and HRQoL remains limited. For instance, one of the reviews (Engelkes et al., 2015) included one single prospective longitudinal clinical study with a small sample size. Another review (Kocks et al., 2018) referenced only two intervention-focused studies to enhance inhalation technique. The third review (Vazquez-Ortiz et al., 2020) exclusively referenced a cross-sectional study assessing HRQoL, which found no significant outcome differences between patients based on the inhalation technique. This highlights the need for further comprehensive research to fully understand the impact of inhalation technique on various asthma-related outcomes. The lack of a statistically significant association between inhalation technique and the other outcomes of our study deserves further research.

### 4.3 Frequency of reliever use

Our findings about the association of the frequent use of reliever medications with uncontrolled asthma symptoms and exacerbation occurrence align with those from the Nivel Primary Care Database from the Netherlands (Vervloet et al., 2022), which also observed them. Two studies conducted across European countries (Quint et al., 2022) and Canada (Noorduyn et al., 2022) also reported the association between the use of SABA and exacerbations occurrence. Furthermore, our study revealed an association between frequent reliever use and worse HRQoL, a relationship that has been explored less. A cross-sectional analysis of the study in France and the United Kingdom measuring the impact of asthma (Hernandez et al., 2018) showed statistically significant differences of HRQoL, according to the frequency of reliever medication use; among women, those using reliever medication almost or every day presented the biggest deviation from the reference norms.

Our findings suggest that the frequent use of reliever medication, which potentially reflects a reactive approach to asthma management, negatively impacts HRQoL. This observation ties in with our earlier hypothesis regarding adherence and inhalation technique, where proactive self-management practices are contrasted with reactive behaviors. Such patterns underline the complex dynamics of asthma self-management and emphasize the need for future research to conduct a more in-depth exploration of the within-person fluctuations in reliever use and its impact on HRQoL.

### 4.4 Asthma symptom control

The positive long-term association between asthma symptom control and HRQoL found in our study was consistent with a longitudinal study in dyads of asthmatic children and their parents in USA (Li et al., 2017), showing that poorly controlled asthma status was associated with poor HRQoL. Additionally, a systematic review on adolescents (Vazquez-Ortiz et al., 2020) identified poor disease control, exacerbations, and asthma severity as the main factors associated with impaired HRQoL. In contrast, the longitudinal Dutch study in adolescents (Tiggelman et al., 2015) found that higher HRQoL at baseline did not predict changes in asthma control over time. On the other hand, the lower risk of exacerbations among patients with better asthma symptom control observed in our study aligns with the Asthma Care logic process model (Dima et al., 2016) and the GINA guideline (Global Initiative for Asthma, 2023), which position asthma control as directly related to exacerbations.

### 4.5 Sociodemographic factors

Our research identified gender differences in asthma outcomes, with girls experiencing worse asthma symptom control and HRQoL compared to boys. This finding is supported by literature reviews (Vazquez-Ortiz et al., 2020; Chowdhury et al., 2021; Jenkins et al., 2022) that also show an association of HRQoL and asthma control impairment with the female gender. Additionally, we observed that individuals aged 12 years and older showed a decreased HRQoL. These associations could be attributed to hormonal changes impacting airway inflammation, potential variances in immune responses, and the distinctive psychosocial challenges faced by female subjects and adolescents, as previously explained (de Benedictis and Bush, 2017; Chowdhury et al., 2021; Jenkins et al., 2022). These factors might collectively contribute to worsened asthma symptoms and treatment outcomes, subsequently affecting HRQoL. Nevertheless, the Dutch study in adolescents (Tiggelman et al., 2015) observed an increase in adolescents’ HRQoL over time, attributing this to the possibility that they may perceive their illness as less of a concern. Furthermore, we found that a lower risk of exacerbations was associated with a higher age, which could be related to fewer virus-induced exacerbations, since they are more common in younger children (Ramsahai et al., 2019).

### 4.6 Limitations

Interpreting our findings requires taking into account various limitations. First, we did not consider the interplay of other important factors, such as comorbidities (rhinitis, obesity, and anxiety among others) and environmental triggers. Second, our results do not preclude the potential benefits of a deliberate effort to improve the overall adherence and inhalation technique due to the participation in a study, which could potentially impact their relationship with outcomes and the outcomes themselves. Third, the InTeQ’s reliance on a long recall period (previous 6 months) introduces a potential recall bias. Fourth, the measurement of adherence and the inhalation technique is based on the patient or proxy reporting. Thus, future research could benefit from pharmacy claims, performance tests, and smart inhalers for studying these complex relationships.

Finally, our analysis did not differentiate among specific LABA drugs in the ICS fixed-dose combination treatments (Global Initiative for Asthma, 2023) nor between the types of inhaler devices. Unfortunately, our sample size misbalance among the treatments used (712 reports of ICS-salmeterol, 124 ICS-vilanterol, and 69 ICS-formoterol; 226 patients used pMDI vs. 59 using DPI) prevented carrying out stratified analysis to explore the differences. However, the associations of LABA drugs and the type of inhaler device with adherence were not statistically significant (data not shown). Differences were only found between both inhaler devices among the patients presenting good inhalation technique (41.6% with pMDI vs. 60.7% with DPI, *p* = 0.002), as expected, since children are most likely to use pMDI with a spacer. Therefore, the impact of different inhaler devices on the association between inhaler technique and clinical outcomes merits further research.

## 5 Conclusion

To the best of our knowledge, this is the first longitudinal study specifically conducted in pediatric patients to assess both HRQoL and clinical outcomes (asthma symptom control and exacerbation occurrence), allowing for the evaluation of their longitudinal relationships with two of the main indicators of the quality use of inhalers (i.e., adherence and the inhalation technique). Methodologically, the hierarchical mixed model approach adopted has the advantage of describing how each person changes over time (within-person) and how these changes differ across people (between-person). In addition, conceptually, the timelines–events–objectives–sources (TEOS) framework (Li et al., 2017) has been applied to operationalize adherence.

Our findings highlight the multifaceted nature of asthma in children and adolescents, getting closer to a comprehensive understanding of the dynamic process of asthma treatment and outcomes over time. It is remarkable how although treatment adherence showed to be excellent, a third of the participants reported a suboptimal inhalation technique, supporting the need of actions for improvement in the asthma management of pediatric population. We found longitudinal associations at the within-person level of the two indicators of quality use of inhalers: for adherence to ICS-based maintenance treatment with symptom control and HRQoL, as well as for the inhalation technique with HRQoL. This reinforces the importance of further examining changes over time alongside the changes across people. Notably, the frequency of reliever use was associated with symptom control, exacerbation occurrence, and HRQoL; this pointed out the need for examining within-person changes in reliever use, which is further than the usually assessed between-person differences. Finally, due to the differences observed between boys and girls, it is especially important to apply a gender perspective in clinical practice and future studies on children and adolescents with asthma.

## Data Availability

The raw data supporting the conclusion of this article will be made available by the authors, without undue reservation.

## Abbreviations

ICS, inhaled corticosteroids; ICS plus LABA, inhaled corticosteroids plus long-acting beta-agonist; ICC, intra-class correlation coefficient; VPC, variance partition coefficient; AIC, Akaike information criterion; BIC, Bayesian information criterion.
